# Changes in the asymmetric distribution of cholesterol in the plasma membrane influence streptolysin O pore formation

**DOI:** 10.1038/s41598-019-39973-x

**Published:** 2019-03-14

**Authors:** Fumihiko Ogasawara, Fumi Kano, Masayuki Murata, Yasuhisa Kimura, Noriyuki Kioka, Kazumitsu Ueda

**Affiliations:** 1 Graduate School of Agriculture, Kyoto University, Kyoto, 606-8502 Japan; 2 Institute of Innovative Research, Tokyo Institute of Technology, Kanagawa, 226-8503 Japan; 3 Graduate School of Arts and Sciences, The University of Tokyo, Tokyo, 153-8902 Japan; 4 Institute for integrated Cell-Material Sciences (iCeMS), Kyoto University, Kyoto, 606-8502 Japan

## Abstract

ATP-binding cassette A1 (ABCA1) plays a key role in generating high-density lipoprotein (HDL) and preventing atherosclerosis. ABCA1 exports cholesterol and phospholipid to apolipoprotein A-I (apoA-I) in serum to generate HDL. We found that streptolysin O (SLO), a cholesterol-dependent pore-forming toxin, barely formed pores in ABCA1-expressing cells, even in the absence of apoA-I. Neither cholesterol content in cell membranes nor the amount of SLO bound to cells was affected by ABCA1. On the other hand, binding of the D4 domain of perfringolysin O (PFO) to ABCA1-expressing cells increased, suggesting that the amount of cholesterol in the outer leaflet of the plasma membrane (PM) increased and that the cholesterol dependences of these two toxins differ. Addition of cholesterol to the PM by the MβCD–cholesterol complex dramatically restored SLO pore formation in ABCA1-expressing cells. Therefore, exogenous expression of ABCA1 causes reduction in the cholesterol level in the inner leaflet, thereby suppressing SLO pore formation.

## Introduction

ATP-binding cassette A1 (ABCA1) is ubiquitously expressed in the body and plays a key role in the generation of high-density lipoprotein (HDL)^1–3^. ABCA1 loads cholesterol and phosphatidylcholine (PC) onto a lipid acceptor apolipoprotein A-I (apoA-I) in serum to generate discoidal nascent HDL^4^. Recent work suggested that ABCA1 is associated with other various cellular events, e.g., modulation of growth signaling, adaptation to cell crowding, and inflammatory responses of macrophages^5–7^. However, because ABCA1-mediated HDL generation is regulated at the transcriptional level, and the bloodstream maintains a level of ~5 μg/ml lipid-free apoA-I^8^, ABCA1-mediated apoA-I–dependent HDL generation is not a sufficiently fast and tunable reaction to regulate these cellular events. When excess cholesterol accumulates in cells, intracellular concentrations of oxysterols increase; subsequently, the liver X receptor (LXR), activated via binding of oxysterols, stimulates transcription of *ABCA1*. Because ABCA1 is a large membrane protein, consisting of 2261 amino acid residues, its transcription, splicing, translation, and maturation require several hours after transcriptional activation.

Recently, we reported that cholesterol is asymmetrically distributed in the PM, and that this cholesterol distribution modulates Wnt3a signaling^5^, although this remains controversial^9^. Under normal conditions, cholesterol concentration in the outer leaflet is higher than in the inner leaflet, and the asymmetric distribution disappears when both ABCA1 and ABCG1 are knocked down and sphingomyelin (SM) in the PM is degraded by sphingomyelinase (SMase). On the other hand, Courtney *et al*.^10^ reported that cholesterol primarily (80%) resides in the cytoplasmic leaflet in the PM of human erythrocytes. Because cholesterol is not synthesized in erythrocytes, excess cholesterol does not accumulate in erythrocytes; it is likely that ABCA1 does not function in these cells. Furthermore, because erythrocytes do not receive external stimuli, such as growth signals, modulation of signaling by cholesterol distribution is not necessary. We speculate that cholesterol distribution in the PM could differ between erythrocytes and other body cells. These results suggest that ABCA1 continuously flops cholesterol from the inner to the outer leaflet of the PM in cells other than erythrocytes, in which SM maintains the cholesterol gradient, and that it regulates cellular events via this activity.

Streptolysin O (SLO) is a member of the cholesterol-dependent cytolysin (CDC) family, a large family of pore-forming toxins. It has been proposed that CDCs initially bind to the PM via cholesterol and form oligomerized complexes, and are then inserted into the membrane and create β-barrel pores^11^. However, although SLO forms a pore dependent upon cholesterol in the PM, SLO binds to the PM even after cholesterol depletion^12^, and a SLO mutant in which two residues in the cholesterol-binding domain have been replaced also binds to the PM^12,13^. Therefore, it remains unclear how SLO binds to the PM and forms a pore.

Because the pore formed by SLO is large enough for cytosolic proteins (or any proteins of interest) to pass through, SLO can be used for semi-intact cell experiments^14,15^. We tried to establish the conditions for semi-intact cell experiments using SLO, with the goal of revealing the mechanism of the function of ABCA1. Unexpectedly, however, we found that ABCA1-expressing cells could barely be permeabilized by SLO. In light of the cholesterol transport activity of ABCA1 and the cholesterol dependency of SLO pore formation, we speculated that a change in cholesterol distribution could be associated with suppression of SLO pore formation. In this study, we tested the hypothesis that ABCA1 changes the cholesterol distribution in the PM, thereby suppressing SLO pore formation.

## Results

### ABCA1 inhibits pore formation by SLO in an ATPase-dependent manner

**Figure 1:**
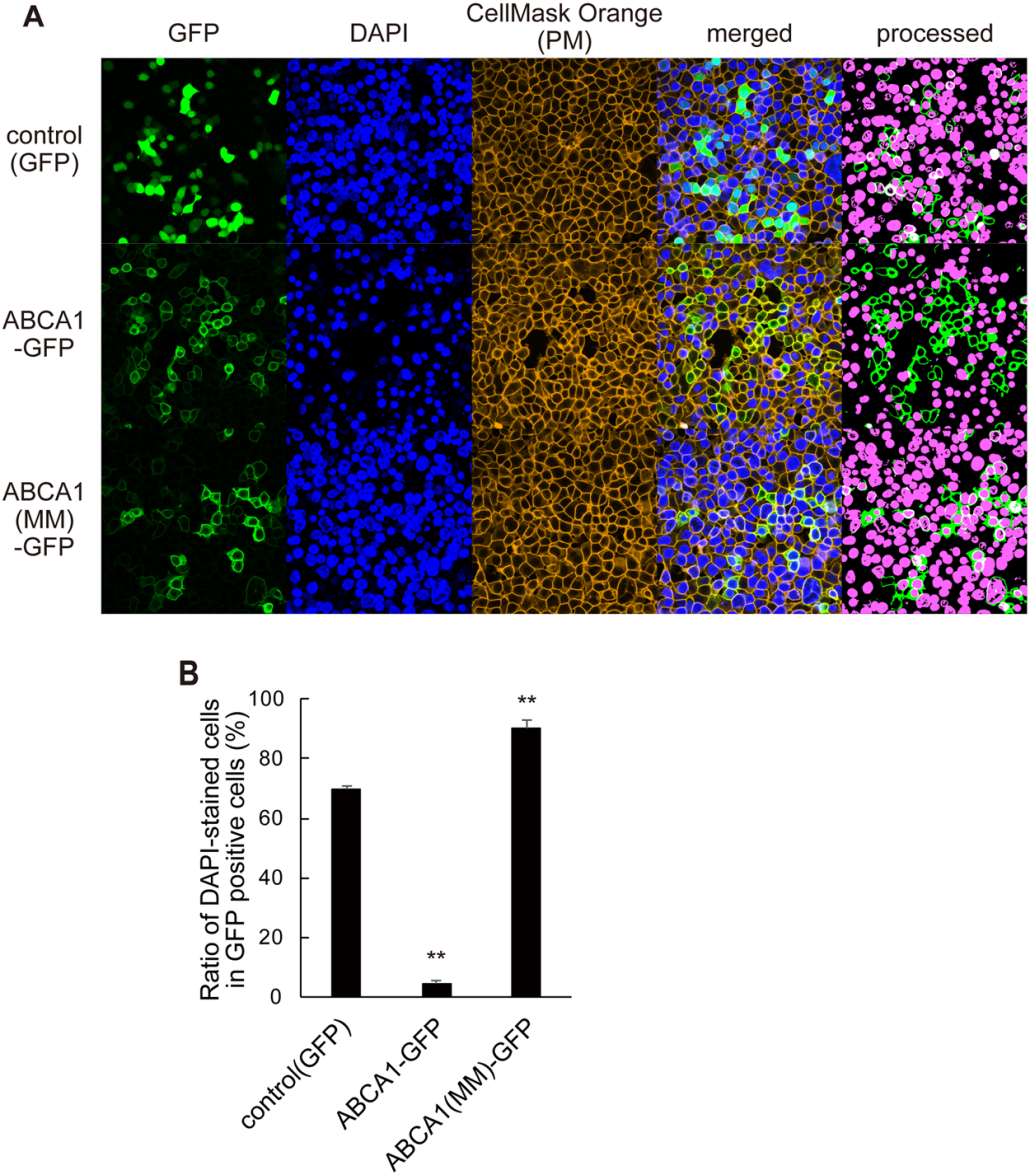
ABCA1 inhibits pore formation by SLO in an ATPase-dependent manner. (**A**) HEK293 cells transiently transfected with plasmids encoding GFP, ABCA1-GFP, and ABCA1(MM)-GFP were treated with SLO and DAPI. To make images clearer for counting DAPI-stained and GFP-positive cells, the images were processed (panel at the right end) as described in Methods. **(B)** DAPI-stained (magenta in processed image) and unstained GFP-positive cells were counted, and proportions were calculated. Average values are shown with S.E. For each samples, images were acquired at five positions in the dish. **P < 0.001 vs. control (GFP).

To examine the effect of ABCA1 on SLO pore formation, cells expressing GFP, ABCA1-GFP, and non-functional ATPase-deficient ABCA1(MM)-GFP were treated with SLO and DAPI. Although nuclei were efficiently stained with DAPI in cells expressing GFP or ABCA1(MM)-GFP, very little DAPI staining was observed in ABCA1-GFP–expressing cells (see processed images of Fig. 1A). The proportion of DAPI-positive cells in ABCA1-GFP–expressing cells was 4.5%, whereas in control GFP-positive and ABCA1(MM)-GFP–positive cells, the proportions were greater than 70% (Fig. 1B). Because DAPI does not pass through the intact membrane, only nuclei of cells in which the plasma membrane was permeabilized by the pore-forming action of SLO were stained. These results indicate that ABCA1 makes cells resistant to the pore-forming action of SLO, and that this property is ATPase-dependent.

### SLO binding is not affected by ABCA1

**Figure 2:**
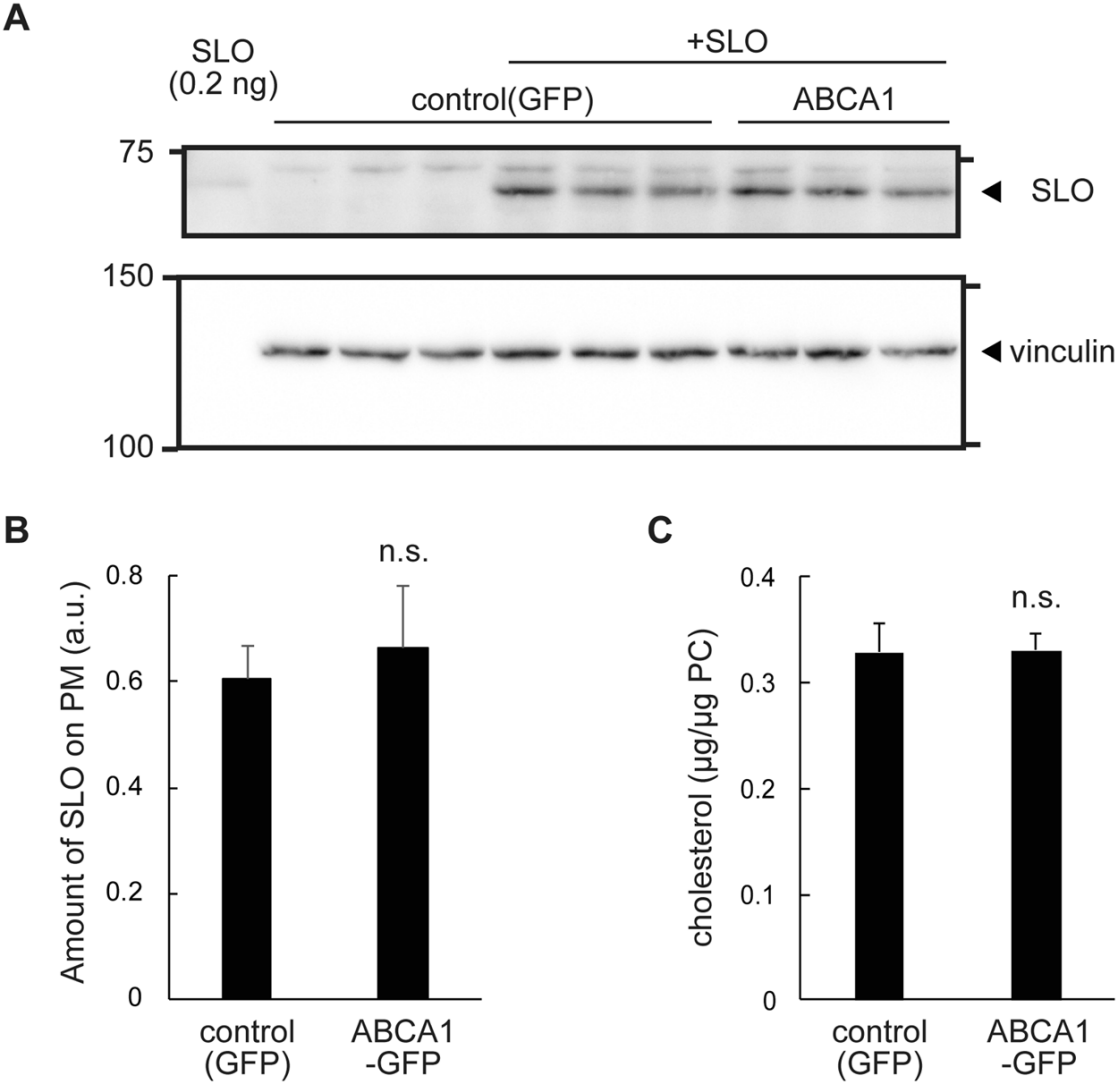
SLO binding and cholesterol content in the PM are not affected by ABCA1. (**A**) HEK293 cells transiently transfected with plasmids for GFP and ABCA1-GFP were lysed soon after treatment with SLO and analyzed by western blotting with anti-SLO and anti-vinculin antibodies; the latter was used as a loading control. (Original images are shown in Supplementary Fig. 4.) (**B**) SLO band intensity was measured using Fiji software and normalized against the vinculin band intensity in the same membrane. The experiment was performed in triplicate, and average values are shown with S.E. n.s., P > 0.05 vs. control (GFP). (**C**) Cholesterol content in membranes. Membrane fractions were isolated from HEK293 cells transfected with plasmids for GFP and ABCA1-GFP and cultured under the same conditions used for the SLO pore formation experiment. The cholesterol content was normalized against the phosphatidylcholine content from the same sample. The experiment was performed in triplicate, and average values are shown with S.E. n.s., P > 0.05 vs. control (GFP).

Because SLO is a member of the cholesterol-dependent cytolysin family, we hypothesized that ABCA1 causes a change in the cholesterol content of the PM, thereby reducing binding of SLO to the PM. Unexpectedly, however, there was no significant difference in the amount of SLO bound between cells expressing ABCA1-GFP and GFP (Fig. 2A,B). Because cells were incubated with SLO in the absence of apoA-I, which required for HDL generation, cholesterol export from cells was not expected to occur. Indeed, free cholesterol content in the total membrane fraction was not altered by ABCA1 expression (Fig. 2C). These results suggest that ABCA1 suppresses the pore-formation step, but not the binding step, of SLO.

### PFO-D4–accessible cholesterol in the outer leaflet of the PM is increased by ABCA1

**Figure 3:**
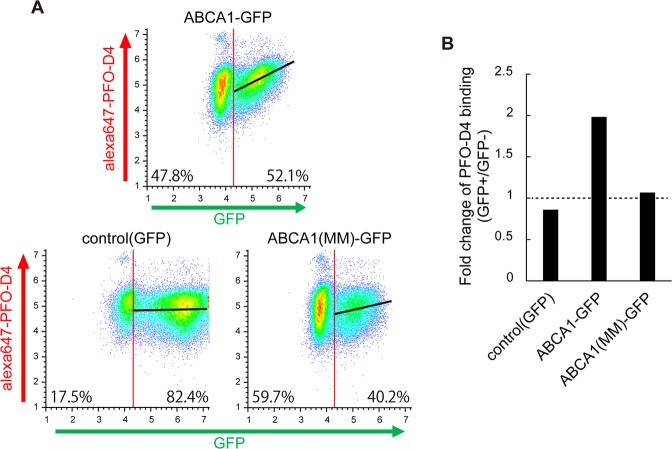
PFO-D4–accessible cholesterol in the outer leaflet of the PM is increased by ABCA1. (**A**) PFO-D4–accessible cholesterol in the outer leaflet of the PM was analyzed by flow cytometry. PFO-D4 was labeled with Alexa Fluor 647. FreeStyle293-F cells with fluorescent intensities greater than 20,000 were defined as GFP-positive, and others as GFP-negative. Linear regression between GFP and Alexa Fluor 647 is shown as a black line. (**B**) Fold change in the median value of Alexa Fluor 647 fluorescence intensity in GFP-positive vs. GFP-negative cells.

It has been proposed that the plasma membrane contains different types or pools of cholesterol, e.g., lipid raft and non-raft^16^ or labile and non-labile^17^. It is likely that the cholesterol content in the PM was not altered, as the removal of cholesterol from cells did not occur, and free cholesterol content in the total membrane fraction was unaffected by expression of ABCA1 (Fig. 2C). We therefore speculated that the distribution or organization of cholesterol in the PM could be affected by ABCA1. To explore this possibility, we performed flow cytometry using the Alexa Fluor 647–labeled D4 domain of perfringolysin O (PFO), which binds to cholesterol in the outer leaflet of the PM^5,18^. FreeStyle293-F suspension cells were used in this assay to obviate the necessity of trypsin treatment, which digests ABCA1 on the cell surface^4^. PFO-D4 binding increased with expression of ABCA1: the median value was about 2-fold higher in the ABCA1-GFP–positive population, but was not much changed in the control GFP–positive cells or ABCA1(MM)-GFP–positive cells (Fig. 3A,B). These results suggest that the level of PFO-D4–accessible cholesterol in the outer leaflet of the PM is increased by exogenous expression of ABCA1.

### MβCD-cholesterol treatment drastically increases SLO pore formation

**Figure 4:**
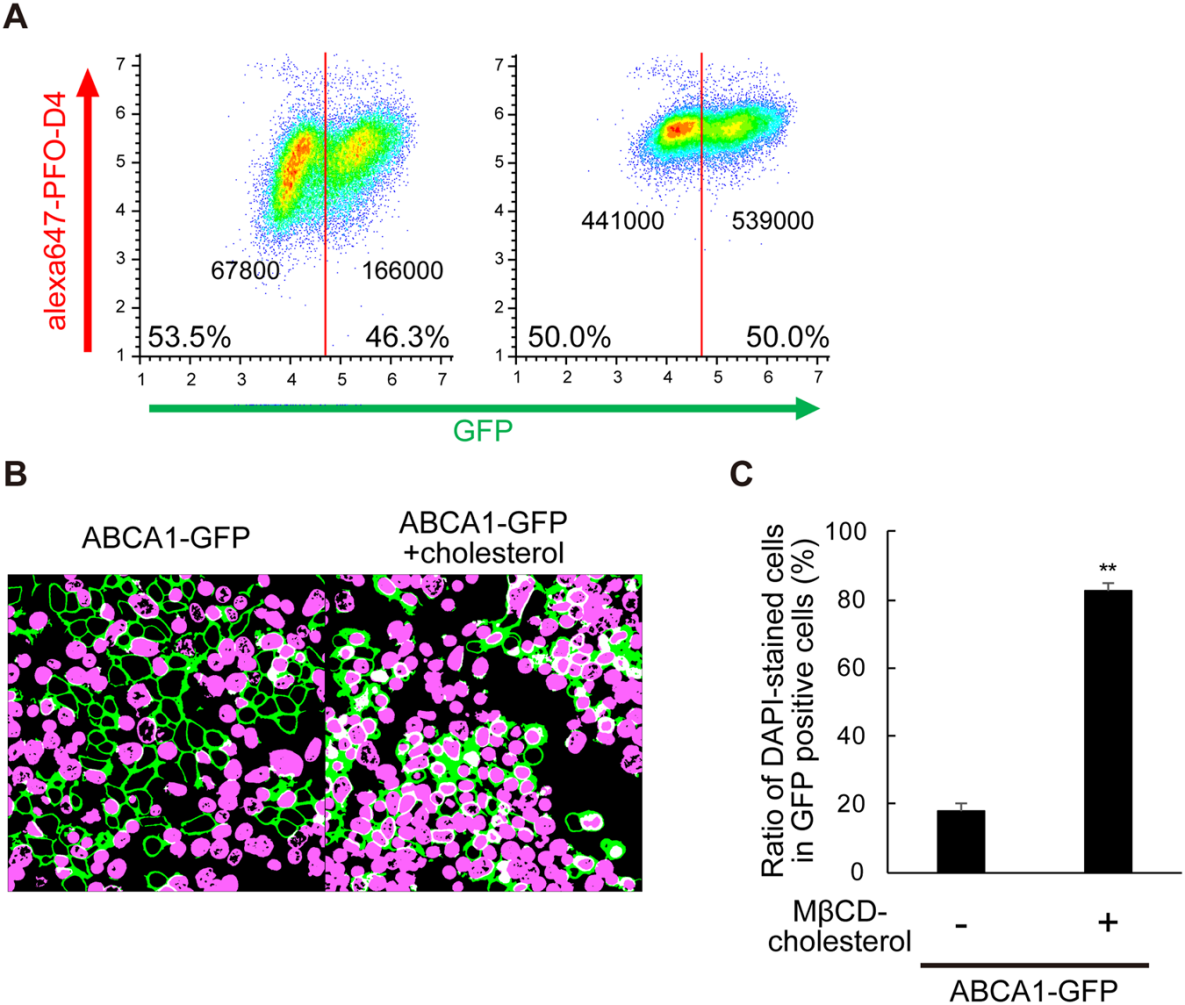
MβCD-cholesterol treatment drastically increases SLO pore formation. (**A**) FreeStyle293-F cells transiently transfected with plasmids encoding ABCA1-GFP were treated with (right) or without (left) MβCD–cholesterol complex just before flow cytometry analysis. Cells with fluorescence intensity greater than 50,000 were defined as ABCA1-GFP–positive, and others as ABCA1-GFP–negative. The median value of Alexa Fluor 647 fluorescence intensity of each population is shown. (**B**) HEK293 cells transiently transfected with plasmids encoding ABCA1-GFP were treated with (right) or without (left) MβCD–cholesterol complex just before the SLO treatment (original images are shown in Supplementary Fig. 1). (**C**) DAPI-stained and -unstained cells in GFP-positive cells were counted, and proportions were calculated. Average values are shown with S.E. For each sample, images were acquired at five positions in the dish. **P < 0.001 vs. cells not treated with MβCD–cholesterol complex.

Next, we examined the effect of treatment with the MβCD–cholesterol complex, which would increase the level of cholesterol in the PM. This treatment strongly increased PFO-D4 binding to both ABC1-GFP-negative and positive cells (Fig. 4A) and the ratio of DAPI-positive cells in ABCA1-GFP–expressing cells was dramatically restored, from 18% to 83% (Fig. 4B,C), suggesting that SLO pore formation efficiently occurred in ABCA1-GFP–expressing cells after the addition of cholesterol. The PFO-D4–accessible cholesterol level in the outer leaflet was also increased in cells in which SLO pore formation was suppressed by ABCA1 (Fig. 3). These results suggest that the suppression of SLO pore formation was not due to a change in cholesterol level or organization in the outer leaflet of the PM but to a change in cholesterol content of the inner leaflet.

### SMase treatment increases SLO pore formation in control cells, but not in ABCA1-expressing cells

**Figure 5:**
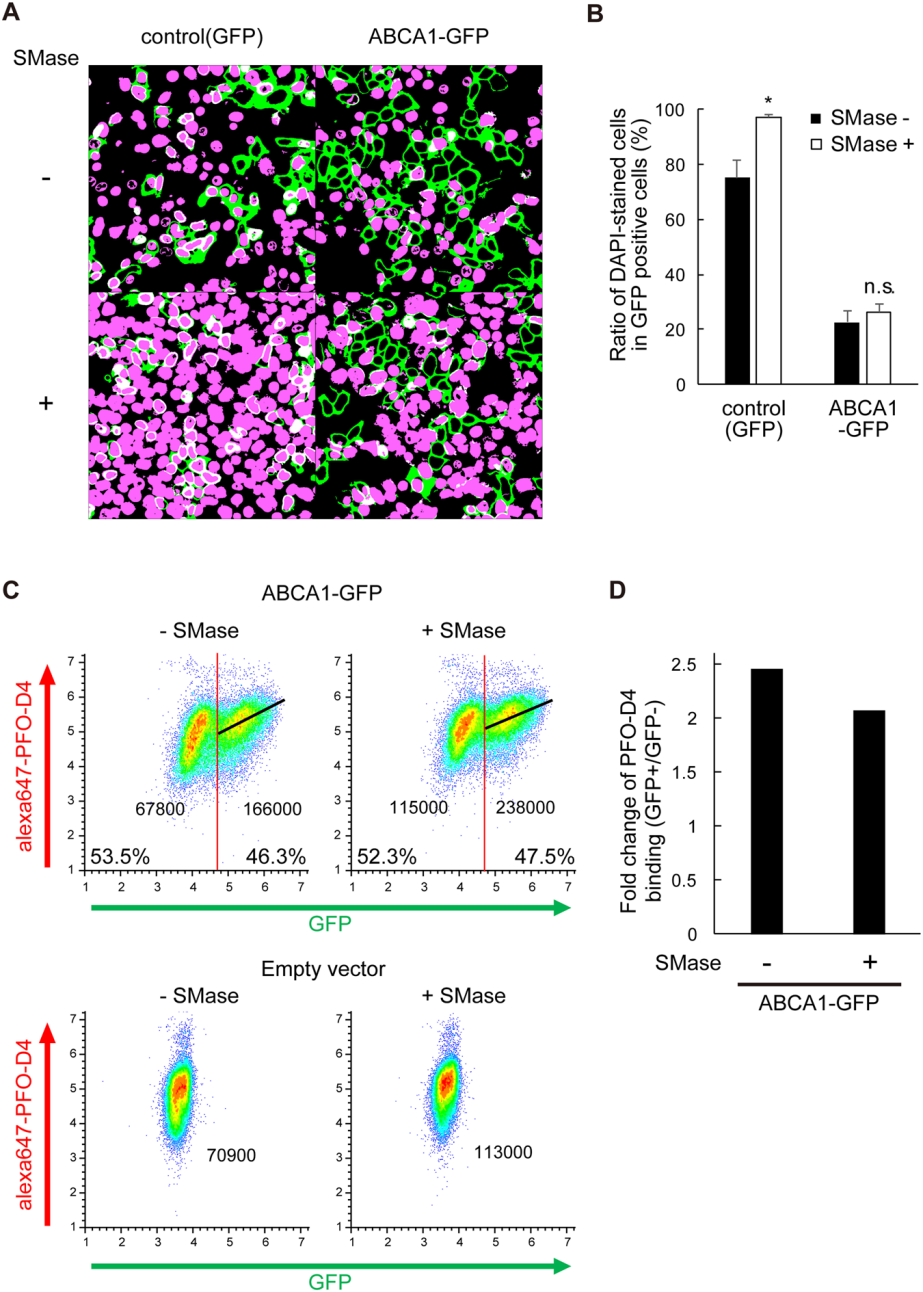
SMase treatment increases SLO pore formation in control cells, but not in ABCA1-expressing cells. (**A**) HEK293 cells transiently transfected with plasmids for GFP and ABCA1-GFP were treated with SMase just before the treatment with SLO and DAPI (original images are shown in Supplementary Fig. 2). (**B**) DAPI-stained and -unstained GFP-positive cells were counted, and proportions were calculated. Filled bars, before SMase treatment; empty bars, after SMase treatment. Average values are shown with S.E. For each sample, images were acquired at five positions in the dish. *P < 0.01 compared with SMase-untreated. n.s. P > 0.05 compared with control (GFP). **(C)** FreeStyle293-F cells transiently transfected with plasmids encoding ABCA1-GFP or control empty vector were treated with SMase just before flow cytometry analysis. Cells with fluorescence intensity greater than 50,000 were defined as GFP-positive, and others as GFP-negative. Linear regression between GFP and Alexa Fluor 647 is shown as a black line. The median value of Alexa Fluor 647 fluorescence intensity of each population is shown. (**D**) Fold change in the median value of Alexa Fluor 647 fluorescence intensity in GFP-positive vs. GFP-negative cells.

Next, we examined the effect of treatment with SMase, which was expected to perturb cholesterol pools in the PM by degrading SM^5,17^. In control cells, the ratio of DAPI-positive cells increased from 75 ± 2.0% to 97 ± 1.3% following treatment (Fig. 5A,B). By contrast, the ratio of DAPI-positive cells did not increase much after treatment of ABCA1-GFP–expressing cells (22 ± 4.1% and 26 ± 3.5% before and after treatment, respectively) (Fig. 5A,B). PFO-D4–binding increased in both ABCA1-negative and positive cells, and also in control cells, and the median value in the ABCA1-GFP–positive population was 2-fold higher than in the ABCA1-GFP–negative population, even after SMase treatment (Fig. 5C,D). SMase treatment releases cholesterol from SM-associated pool and increases labile SM-free cholesterol both in the outer and inner leaflet of the PM. Therefore, our results suggest that the suppression of SLO pore formation is due to a change in cholesterol content in the inner leaflet and that exogenously expressed ABCA1 maintains cholesterol content in the inner leaflet even in the absence of SM.

### ABC proteins, which transport cholesterol, inhibit SLO pore formation

**Figure 6:**
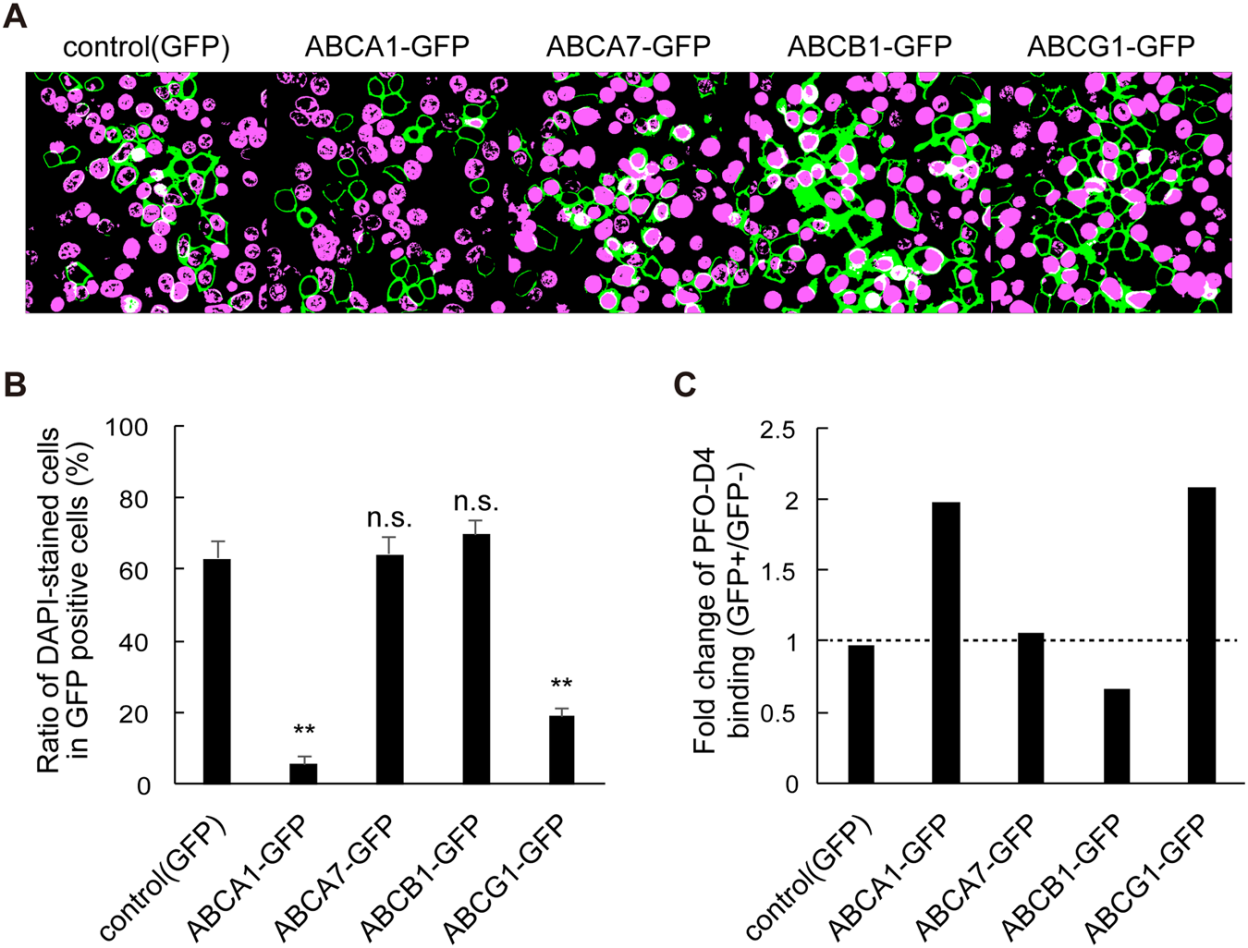
Cholesterol-transporting ABC proteins inhibit SLO pore formation. (**A**) HEK293 cells transiently transfected with plasmids encoding GFP, ABCA1-GFP, ABCA7-GFP, ABCB1-GFP, or ABCG1 were treated with SLO and DAPI (original images are shown in Fig. S3). (**B**) DAPI-stained and -unstained GFP-positive cells were counted, and proportions were calculated. Average values are shown with S.E. For each sample, images were acquired at five positions in the dish. **P < 0.001 compared with control (GFP). n.s. P > 0.05 compared with control (GFP). (**C**) Cholesterol content in the outer leaflet of the PM was analyzed by FACS with PFO-D4 labeled with Alexa Fluor 647. FreeStyle293-F cells with fluorescence intensity greater than 50,000 were defined as GFP-positive, and others as GFP-negative. Original data are shown in Supplementary Fig. 3.

Finally, we examined other ABC proteins, which transport hydrophobic substrates including cholesterol, to demonstrate that ABCA1 inhibited SLO pore formation by moving cholesterol as a substrate (Fig. 6). ABCG1, which transports cholesterol to HDL^19^ and contributes to the asymmetric distribution of cholesterol in the PM together with ABCA1^5^, inhibited SLO pore formation, just as ABCA1 did. The proportions of DAPI-positive cells were 19% and 5.5% in ABCG1-GFP– and ABCA1-GFP–expressing cells, respectively. On the other hand, in cells expressing ABCA7, a close homolog of ABCA1 that transports phosphatidylcholine in an apoA-I–dependent manner, but does not transport cholesterol^20^, the proportion of DAPI-positive cells (64%) was as high as in control GFP cells (63%). In cells expressing ABCB1, a multidrug transporter that does not transport cholesterol^21^, the proportion was 70%. Among these ABC proteins, only ABCA1 and ABCG1 increased PFO-D4 binding (Fig. 6C). These results suggest that the inhibitory effect on SLO pore formation is dependent upon cholesterol transport activity.

## Discussion

**Figure 7:**
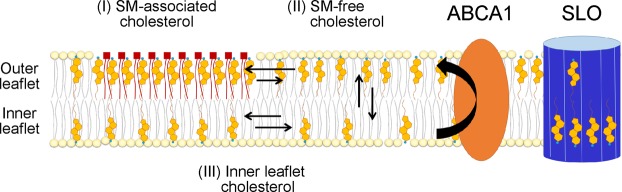
Schematic illustration of cholesterol distribution in the PM. The PM contains three different types or pools of cholesterol: (I) SM-associated cholesterol in the outer leaflet; (II) SM-free cholesterol in the outer leaflet; and (III) cholesterol in the inner leaflet. ABCA1 flops cholesterol from the inner to the outer leaflet of the PM. The enhanced asymmetric distribution of cholesterol caused by exogenously expressed ABCA1 suppresses SLO pore formation.

In this study, we found that SLO pore formation was suppressed in ABCA1-expressing cells. The amount of SLO bound to the PM was not altered by ABCA1 expression (Fig. 2), suggesting that the pore-forming step of SLO after binding to the PM was suppressed. The level of PFO-D4–accessible cholesterol in the outer leaflet of the PM was increased by ABCA1 (Fig. 3), whereas the free cholesterol content in the total membrane fraction was not altered (Fig. 2C). The addition of cholesterol to the PM restored SLO pore formation in ABCA1-GFP–expressing cells (Fig. 4). It has been proposed^17^ that the PM contains three different types or pools of cholesterol: (1) a pool accessible to the bacterial toxin PFO, which binds cholesterol in membranes; (2) a SM-sequestered pool that binds PFO only after SM is degraded by SMase; and (3) a residual pool that does not bind PFO even after SMase treatment. If we add to this picture the concept of transbilayer distribution in the two leaflets of the PM, we can reconsider these pools as follows (Fig. 7): (I) SM-associated cholesterol in the outer leaflet; (II) SM-free cholesterol in the outer leaflet; and (III) cholesterol in the inner leaflet. Treatment with MβCD–cholesterol complex, which increased PFO-D4 binding to cells, dramatically increased SLO pore formation (Fig. 4), and the PFO-D4–accessible cholesterol level was also increased in cells in which SLO pore formation was suppressed by ABCA1 (Fig. 3), suggesting that the suppression of SLO pore formation was not due to a change in pool-II cholesterol. SMase treatment, which increases pool-II and pool-III cholesterol, increased SLO pore formation in control cells, but not in ABCA1-GFP–expressing cells (Fig. 5). Landry *et al*.^22^ has reported that there is no obvious difference in terms of plasma membrane total cholesterol content in ABCA1 and mock-transfected cells and that MβCD can extract 40–50% more cholesterol from ABCA1 cells than from mock-transfected cells at 0 °C, suggesting that ABCA1 expression increases the level of cholesterol in the outer leaflet of the PM. This indicates that increase in cholesterol level in the outer leaflet by ABCA1 is a cause of increased PFO-D4 binding observed in this study. These results suggest that a change in the content of pool-III cholesterol affects SLO pore formation. Because ABCA1 suppresses SLO pore formation even after SMase treatment, it is likely that ABCA1 maintains the content of pool-III cholesterol even in the absence of SM.

The transverse asymmetry (sidedness) of cholesterol in plasma membrane bilayers became a hot topic recently^23–25^. Several studies reported that cholesterol and ergosterol concentrations in the inner leaflet are 2–4-fold higher than in the outer leaflet^10,26,27^, whereas Liu *et al*.^5^ reported that cholesterol concentration is higher in the outer leaflet than in the inner leaflet. Although it is unclear what caused these discrepancies, it could be due to differences in how cholesterol distribution was analyzed and which pools of cholesterol were measured. Courtney *et al*.^10^ reported that only 20% of the erythrocyte cholesterol was extracted by MβCD at 0 °C, implying that cholesterol primarily (80%) resides in the cytoplasmic leaflet in the PM of human erythrocytes. However, Zha’s group has also reported that cholesterol in the PM of ABCA1-expressing cells is more accessible to MβCD extraction at 0 °C^22^. These results suggest that ABCA1 flops cholesterol from the inner to the outer leaflet of the PM in cells other than erythrocytes.

Furthermore, it remains unclear how fast cholesterol flip-flops (diffuses between two leaflets of the PM) in living cells, but it has been suggested to proceed on time scales of less than a second^28,29^. However, because the cell membrane contains a high concentration of protein, that interact with membrane lipids, the proportion of membrane lipid molecules that can move freely may be limited^30^ and the average flip-flop rate of cholesterol in the PM could be slower than we supposed. We reported that ABCA1 KD decreases asymmetric cholesterol distribution from 10 to 5-fold^5^, suggesting that ABCA1 itself contributes to the 2-fold asymmetry. Purified ABCA1 hydrolyzes one to three ATP molecules per second^31–33^. Future studies should be carefully measure the speed of cholesterol flopping in living cells to confirm that it is high enough to maintain an asymmetric distribution of cholesterol. Although we have not directly measured asymmetric cholesterol distribution, we observed the suppression of SLO pore formation by ABCA1 not only in HEK293 but also in HeLa and BHK cells (data not shown), suggesting that this phenomenon is not cell type–specific. It is conceivable that flopping of cholesterol (transport from the inner to outward leaflet) by exogenously expressed ABCA1 causes asymmetric cholesterol distribution in the PM, which makes cells resistant to SLO pore formation.

Among the ABC proteins that transport hydrophobic substrates, ABCA1 and ABCG1 suppressed SLO pore formation, whereas ABCB1 and ABCA7 did not (Fig. 6). ABCG1 exports cholesterol and SM to HDL^19,34,35^, and together with ABCA1 contributes to the asymmetric distribution of cholesterol in the PM^5^. ABCA7 does not transport cholesterol, but it is a close homolog of ABCA1 and transports phosphatidylcholine in an apoA-I–dependent manner^20^. ABCB1 is a multidrug exporter that interacts with cholesterol but does not export it^21,36^. These results suggest that the inhibitory effect of these proteins on SLO pore formation is dependent upon their cholesterol transport activity.

While this study was under revision, Courtney *et al*. reported^9^ that phospholipid head groups and acyl chain saturation of phospholipids impact binding of the cholesterol probe DAN-D4, which Liu *et al*. used to demonstrate the asymmetric cholesterol distribution^5^. Furthermore, DAN-D4 binding was highly sensitive to proteins in the medium^9^. Therefore, the capacity of microinjected DAN-D4 to bind the cytoplasmic leaflet of the PM was speculated to be severely diminished in live cells. However, Liu *et al*. reported the increased binding of the probes to the inner leaflet of the PM of cells, in which ABCA1 and ABCG1 were both knocked down, suggesting that the probes functioned in cells. In this study, we showed that SLO pore formation was not affected by ABCA7, which transports phospholipids but not cholesterol (Fig. 6). The addition of cholesterol by MβCD-cholesterol treatment drastically increased SLO pore formation (Fig. 4), and SMase treatment affected SLO pore formation in control cells but not in ABCA1-expressing cells (Fig. 5). Together, these results suggest that the suppression of SLO pore formation is caused by cholesterol flopping (transport from the inner to the outer leaflet) in the PM by ABCA1 and is not merely due to changes in phospholipid environment, but instead to the reduction in the cholesterol level in the inner leaflet (Fig. 7).

As discussed above, the cholesterol dependences of PFO and SLO are quite different, although both are pore-forming toxins in the CDC family: PFO is highly dependent on cholesterol in the outer leaflet at the step of binding to the PM; whereas SLO is not. Moreover, the results of this study suggest that the pore formation step of SLO is dependent on cholesterol in the inner leaflet. This difference could be based on differences in strategies for infection, in the host cells themselves, or in their evolutionary paths. These toxins represent potentially useful tools for detecting asymmetric distributions of cholesterol in the PM and to reveal the novel physiological functions of cholesterol. Given that asymmetric distribution of cholesterol in the two leaflets of the PM is involved in the modulation of various cellular events, such tools would be of immense value for cell biology.

## Materials and Methods

### Materials

Purified SLO (01–531) and the rabbit anti-SLO antibody (64–001) were purchased from Bio Academia. The mouse anti-vinculin antibody (V9131), MβCD, cholesterol, SMase, and DAPI were purchased from SIGMA. CellMask Orange was purchased from Invitrogen.

### Cell culture

HEK293 cells were grown in a humidified incubator (5% CO_2_) at 37 °C in Dulbecco’s modified Eagle’s medium (DMEM) supplemented with 10% heat-inactivated fetal bovine serum (FBS). FreeStyle 293-F cells were maintained in FreeStyle 293 expression medium containing 5 µg/mL gentamicin at 37 °C under 8% CO_2_.

### Plasmids

Expression vectors for wild-type ABCA1 and ABCA1MM tagged with GFP at the C terminus were generated as previously described^31,37^. Expression vector for ABCB1 was generated as previously described^38^, and GFP was inserted into the C terminus. *ABCA7* and *ABCG1* cDNAs were inserted into pEGFP-N2 (Clontech). The expression vector for PFO-D4-GFP was kindly provided by Dr. Toshihide Kobayashi of the University of Strasburg. GFP was removed from the vector using the In-Fusion HD Cloning Kit (Clontech). The DNA fragment was amplified by PCR with primers 5′-CAGCCATATGGCTAGCAAGGGAAAAATAAA-3′ and 5′-CTAGCCATATGGCTGCCGCG-3′.

### Transfection

HEK293 cells were transfected with 1 µg/mL of each expression vector using 2 µg/mL Polyethyleneimine “MAX” (PolySciences)^35^ in DMEM containing 10% FBS. FreeStyle 293-F cells were transfected with 4 µg/mL of each expression vector using 8 µL/mL 293fectin (Thermo Fisher Scientific) in FreeStyle 293 Expression Medium containing 5 µg/mL gentamicin.

### SLO pore formation

HEK293 cells (5 × 10^5^) were subcultured in a 3.5-cm poly-L-lysine–coated glass-base dish in DMEM containing 10% FBS. After 24 h of incubation, the cells were transfected with each expression vector and incubated for an additional 24 h. The cells were washed with Hank’s Balanced Salt Solution (HBSS) and incubated with CellMask Orange in HBSS containing 0.02% BSA at room temperature for 20 min to stain the PM. After CellMask Orange was removed, SLO (100 ng /mL) in ice-cold DMEM was added, and the cells were incubated on ice for 8 min. The cells were then washed three times with PBS− (phosphate-buffered saline without CaCl_2_ and MgCl_2_), incubated with DAPI in transport buffer (25 mM HEPES-KOH, pH 7.4, 115 mM KOAc, 2.5 mM MgCl_2_) at 37 °C for 5 min, washed twice with transport buffer, and observed under a confocal microscope (ECLIPSE Ti; Nikon). Images were acquired in five locations within each sample. Treatment with methyl-beta-cyclodextrin (MβCD)–cholesterol complex (MβCD: cholesterol = 4.5 mM/mL: 0.5 mM/mL) or SMase (0.2 mU/mL) was performed in DMEM containing 0.02% BSA at 37 °C for 30 min before CellMask Orange staining. To avoid cholesterol diffusion, the cells were incubated with CellMask Orange on ice for 10 min.

### Image processing

Images acquired in the SLO treatment assay were processed using the Fiji software. First, the original images of GFP, CellMask Orange, and DAPI were binarized, and noise was removed based on particle size (1–100 pixels) and circularity (0.5–1). The GFP image was subtracted from the inverted image of Cell Mask Orange to represent GFP on the PM. Because GFP leaked through SLO pores, and GFP fluorescence at the PM was quite low in control cells expressing only GFP, the GFP image was acquired with saturated intensity to improve detection. The images of GFP on the PM and DAPI were merged.

### Flow cytometry analysis

FreeStyle 293-F cells were seeded on 6-well plates at a density of 2 × 10^6^ cells per well, and then transfected with each expression vector. After 24 h of rotation culture, the cells were harvested and suspended in HBSS. The cells were incubated at 20 °C for 30 min with PFO-D4 labeled with Alexa Fluor 647, and then analyzed on a flow cytometer (Accuri C6, BD). SMase (0.2 mU/mL) treatment was performed in FreeStyle 293 expression medium containing 5 µg/mL gentamicin at 37 °C for 30 min, prior to harvest. Plot data were exported to Excel, logarithmically transformed, and calculated by linear regression. The pseudocolor plot graph was generated using Cytospec. For each sample, 30,000 cells were analyzed. Intensities of PFO-D4 binding to GFP-positive and -negative cells were compared with the median values in each population.

### Purification of PFO-D4 and labelling with Alexa Fluor 647

*E*. *coli* strain BL21(DE3) was used for overexpression of PFO-D4. After induction with IPTG, *E*. *coli* cells were harvested and resuspended in PBS−. The cell suspension was sonicated and centrifuged, and PFO-D4 was purified from the supernatant using Profinity IMAC Ni-Charged Resin (BIO-RAD). After the buffer was exchanged using a PD MidiTrap G-25 column (GE Healthcare), PFO-D4 was concentrated with an Amicon Ultra-0.5 3k (Merck) and labeled with Alexa Fluor 647 NHS ester (Thermo Fisher Scientific). After buffer exchange and re-concentration of PFO-D4, an equal volume of glycerol was added, and the sample was stored in −30 °C.

### SLO binding

HEK293 cells were seeded on poly-L-lysine–coated 24-well plates and treated as described for the SLO pore formation assay. After SLO treatment, the cells were lysed with RIPA buffer (20 mM Tris-HCl, pH7.5, 1% Triton X-100, 0.1% SDS, 1% sodium deoxycholate) with EDTA-free protein inhibitor cocktail (complete, Roche) on ice. Laemmli buffer^39^ was added, and the sample was incubated at 98 °C for 5 min. Samples were electrophoresed on 10% SDS-polyacrylamide gels and analyzed by western blotting with anti-SLO and anti-vinculin antibodies.

### Measurement of cholesterol content in membrane fractions

HEK293 cells were subcultured in 6-well plates at a density of 5 × 10^5^ cells/well in DMEM containing 10% FBS. After 24 h of incubation, the cells were transfected with each expression vector and incubated for an additional 24 h. The cells were kept on ice, washed with PBS−, and harvested with a cell scraper in PBS−. The cells were then lysed with 30 strokes in a Dounce homogenizer and spun at 2,000 rpm for 15 min in a HITACHI T15A39–1400 rotor to remove nuclei. To isolate membrane fractions, the supernatant was spun at 15,000 rpm for 30 min at 4 °C in the same rotor. The membrane fractions were resuspended in PBS−, and the choline phospholipid (PL) and cholesterol contents were determined using colorimetric enzyme assays as described previously^40^.

### Statistical analysis

The statistical significance of differences between mean values was evaluated using the unpaired t-test. Multiple comparisons were evaluated using the Tukey test following one-way ANOVA. All experiments were performed at least two times.

## Supplementary information

Supplementary information
