# Supplementary material for: Changes in the asymmetric distribution of cholesterol in the plasma membrane influence streptolysin O pore formation
Source: Sci Rep. 2019 Mar 14;9:4548. doi: 10.1038/s41598-019-39973-x (PMC6418215; doi:10.1038/s41598-019-39973-x)

Supplementary Information for “Changes in the asymmetric distribution of cholesterol in the plasma membrane influence streptolysin O pore formation” by Fumihiko Ogasawara, Fumi Kano, Masayuki Murata, Yasuhisa Kimura, Noriyuki Kioka, and Kazumitsu Ueda

Supplementary Fig. 1. Original images of Fig. 4A.

Supplementary Fig. 2. Original images of Fig. 5A.

Supplementary Fig. 3. Original images of Fig. 6A, B.

Supplementary Fig. 4. The membrane used in Fig. 2A.

Supplementary Figure 1

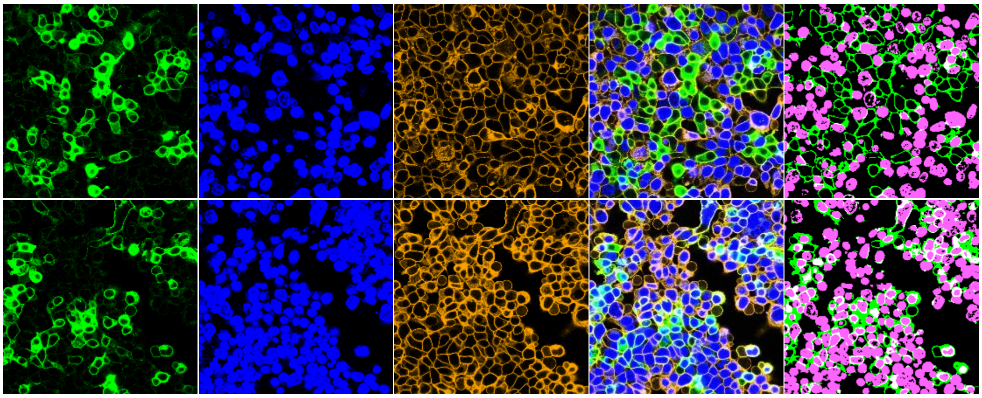

Supplementary Figure 2

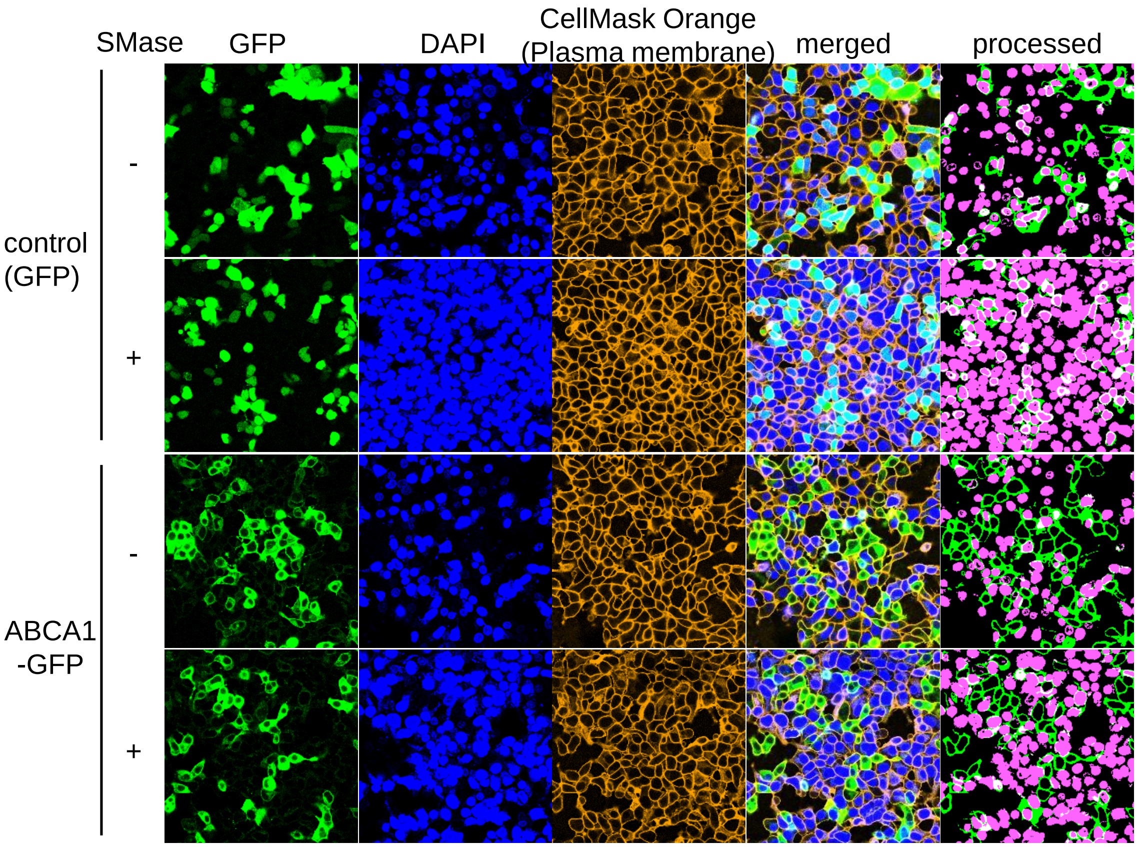

Supplementary Figure 3

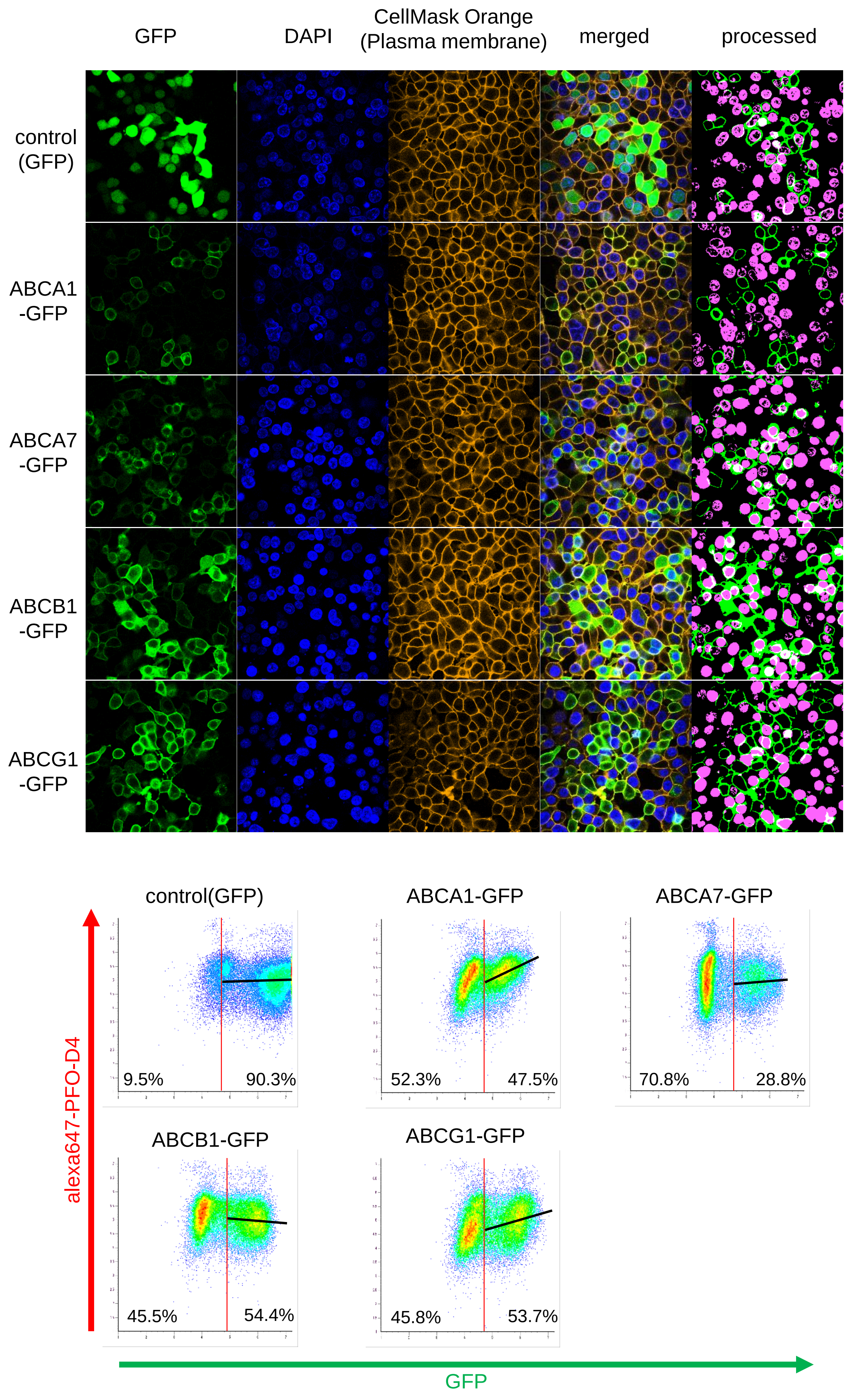

Supplementary Figure 4

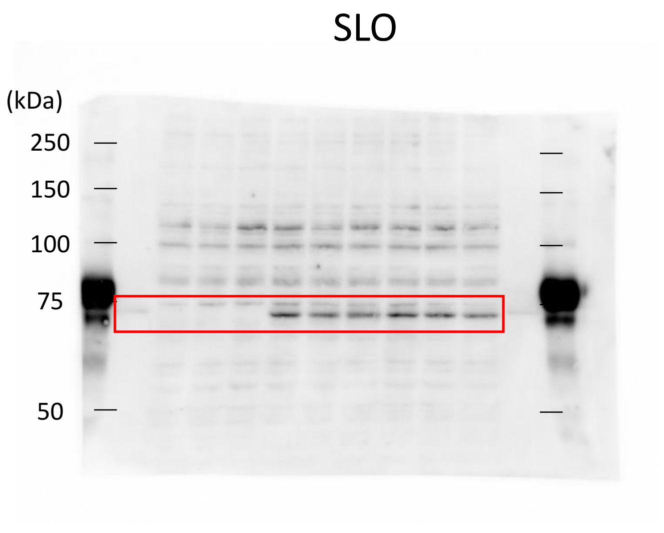

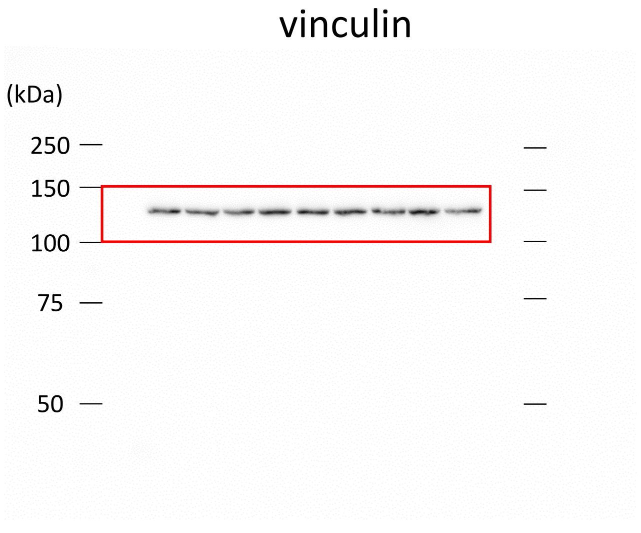

Supplement: Supplementary file 1 — Supplementary information [file 41598_2019_39973_MOESM1_ESM.pdf]
